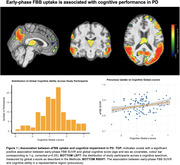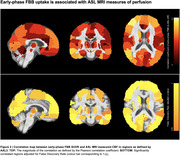# Early phase Amyloid‐PET reproduces metabolic signatures of cognitive decline in Parkinson's disease

**DOI:** 10.1002/alz.091211

**Published:** 2025-01-09

**Authors:** Campbell Le Heron, William Aye, Megan Stark, Kyla‐Louise Horne, Daniel J Myall, Toni Pitcher, Mustafa Almuqbel, Ross Keenan, John C Dalrymple‐Alford, Tim J Anderson, Tracy R Melzer

**Affiliations:** ^1^ University of Otago, Christchurch New Zealand; ^2^ New Zealand Brain Research Institute, Christchurch New Zealand; ^3^ University of Canterbury, Christchurch New Zealand

## Abstract

**Background:**

Establishing practical and effective diagnostic pathways for people with cognitive impairment is a crucial international research priority. Traditional (late‐phase) analysis of an amyloid‐beta (Aβ) PET scan (∼90 minutes after radiotracer injection) provides important information about the presence/absence of underlying Alzheimer’s pathology, but no information about brain metabolism/perfusion. Recent work suggests that amyloid‐beta PET tracer uptake shortly after injection (‘early‐phase’) closely reflects brain metabolism and perfusion. Parkinson’s disease (PD), the second most common neurodegenerative disease worldwide, serves as a model disorder in which cognitive impairment occurs across a spectrum, is not specifically associated with underlying brain amyloid status, and has known patterns of altered metabolism and perfusion associated with cognitive decline. We investigated the utility of early‐phase ^18^F‐Florbetaben Aβ PET (eFBB) uptake as a surrogate marker of cerebral perfusion/metabolism in people with PD. We hypothesised that eFBB uptake would reproduce characteristic patterns of hypometabolism and hypoperfusion associated with cognitive decline in PD, irrespective of late‐phase Aβ status, and positively correlate with a direct measure of cerebral perfusion – ASL MRI.

**Method:**

115 closely phenotyped PD patients across the spectrum of cognitive impairment underwent dual‐phase (early and late) Aβ PET, structural and perfusion (ASL) MRI, and neuropsychological assessments. Multiple linear regression models compared eFBB uptake with 1) cognitive performance and 2) ASL‐MRI perfusion, correcting for multiple comparisons by family‐wise error correction (threshold‐free cluster enhancement).

**Result:**

eFBB uptake significantly correlated with cerebral perfusion across widespread cortical and subcortical regions (r = 0.15‐0.49, p < 0.005, Figure 2). Reduced eFBB uptake was significantly associated with poorer cognitive performance in brain regions previously linked to hypometabolism‐associated cognitive decline in PD: parieto‐occipital regions, middle and inferior temporal gyri, prefrontal cortex and the thalamus (all p < 0.05, Figure 1). These findings were independent of amyloid status.

**Conclusion:**

eFBB uptake is a reliable surrogate measure for cerebral perfusion/metabolism in a predominantly Aβ negative group. It provides complimentary information to the late‐phase Aβ scan, and can add value whether the late phase is positive or negative. Such dual‐phase PET imaging approaches show significant promise in streamlining diagnostic pathways for people with cognitive impairment.